# Neuroendocrine Differentiation of Prostate Cancer Is Not Systematically Associated with Increased 18F-FDG Uptake

**DOI:** 10.3390/diagnostics11030468

**Published:** 2021-03-08

**Authors:** Matteo Bauckneht, Silvia Morbelli, Alberto Miceli, Sara Elena Rebuzzi, Giuseppe Fornarini

**Affiliations:** 1 Nuclear Medicine, IRCCS Ospedale Policlinico San Martino, 16132 Genova, Italy; silviadaniela.morbelli@hsanmartino.it; 2 Department of Health Sciences (DISSAL), University of Genova, 16132 Genova, Italy; albertomiceli23@gmail.com; 3 Medical Oncology Unit 1, IRCCS Ospedale Policlinico San Martino, 16132 Genova, Italy; saraelena89@hotmail.it (S.E.R.); giuseppe.fornarini@hsanmartino.it (G.F.); 4 Department of Internal Medicine and Medical Specialities (Di.M.I.), University of Genova, 16132 Genova, Italy

**Keywords:** metastatic castration-resistant prostate cancer, neuroendocrine differentiation, positron emission tomography, 18F-Fluorodeoxyglucose, 68Ga-Dotatoc

## Abstract

Neuroendocrine differentiation (NED) of prostate cancer represents an acknowledged predictor of resistant and more aggressive disease. NED can be functionally exploited in vivo using PET/CT imaging with somatostatin analogs radiolabeled with 68Ga. Many previous reports have shown that 18F-FDG PET/CT should also be used in cases such as guiding management, as NED is systematically associated with increased glycolysis. We hereby discuss the case of a metastatic prostate cancer patient in which 68Ga-Dotatoc PET/CT revealed the occurrence of NED with low FDG-avidity.

**Figure 1:**
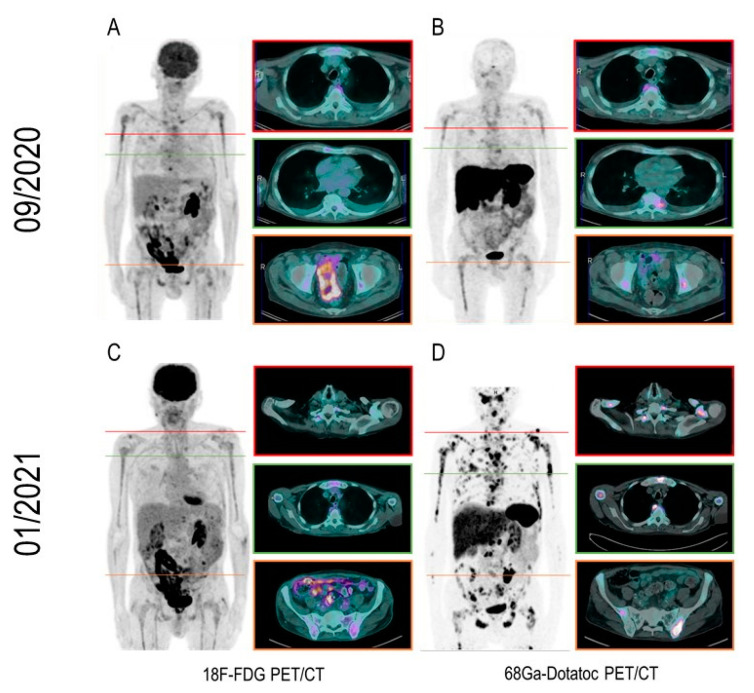
A 69-year-old man was referred to our Institute in June 2012 to diagnose metastatic prostate cancer with a Gleason score of 9 (4 + 5) and lymph nodal and bone involvement since diagnosis. The patient was initially treated with androgen deprivation therapy (LH-releasing hormone analogs and bicalutamide). Since the conversion to castrate-resistant disease (June 2015), the patient had been treated with Abiraterone, Docetaxel, Radium-223, and Cabazitaxel. After three cycles of Cabazitaxel administration, the patient underwent 18F-FDG PET/CT for prognostic purposes. Indeed, while FDG-avidity is low in naïve prostate cancer, it is increased in advanced and heavily pretreated metastatic castration-resistant prostate cancer (mCRPC), providing relevant prognostic insights [1,2,3]. 18F-FDG-PET/CT images of the patient showed low tracer avidity in both lymph nodes and bone lesions, suggesting low glycolytic activity and disease aggressiveness. The PSA level at the time of imaging was 1631 ng/mL. After three further months of chemotherapy, he showed clinical and biochemical progression (subjective increase in pain, PSA level of 2736 ng/mL). However, the subsequent 18F-FDG PET/CT scan showed the disease’s stable metabolic activity (Panel (**A**)). The apparent mismatch between the rising PSA level and the low 18F-FDG-avidity of metastatic lesions raised neuroendocrine differentiation suspicion (NED). While NED represents a relatively rare finding in the initial phases of PC’s natural history (ranging 0.5-2% of total cases), recent data showed that it could reach an incidence of 17-20% in the later stages of the disease (the so-called treatment-emergent neuroendocrine PC, [4,5,6]). This kind of NED represents a later transformation of ordinary adenocarcinoma’s cellular clones to the neuroendocrine phenotype, favored by the selective pressure of androgen-targeted therapy and allowing advanced PC to escape the androgen deprivation [7]. Its main clinical manifestations include androgen deprivation resistance, low PSA levels, the disproportion between PSA kinetics and tumor burden progression, and the eventual increase in serum neuroendocrine tumor markers [8,9]. On this basis, 9 days after, the patient underwent 68Ga-DOTATOC PET/CT (Panel (**B**)), which detected mild tracer uptake in almost all bone lesions seen at 18F-FDG imaging and two 18F-FDG negative pleural lesions. The occurrence of NED was further confirmed by the evidence of moderately increased Chromogranin-A serum levels (122.7 µg/L). These findings guided the subsequent clinical management, as Cabazitaxel was suspended, and the fifth line of systemic therapy with Enzalutamide was then administered to the patient. After three months of Enzalutamide serum, PSA decreased to 833.5 ng/mL. However, a clinical progression was observed. In particular, the Eastern Cooperative Oncology Group performance status (ECOG PS) moved from 0 to 1, as an increase in bone pain and a weight loss of 10 kg were documented. To further explore the apparent mismatch between biochemical and clinical response to Enzalutamide, the patient underwent 18F-FDG and 68Ga-Dotatoc PET/CT within three days for restaging purposes. While the former PET/CT showed very mild to moderate tracer uptake in a few pelvic and vertebral bone lesions (maximum Standardized Uptake Value (SUVmax): 2.5 vs. 4 at baseline), the latter showed a widespread, intensely Dotatoc-avid skeletal, pleural, and lymph nodal disease burden (SUVmax: 64 vs. 5 at baseline) (Panels (**C**,**D**)). This result was interpreted as a progression of the NED part of the disease, not tracked by PSA kinetics in agreement with the acknowledged low-PSA secretion tendency of this kind of tumor [8,9,10]. On the one hand, the present case challenges FDG PET/CT’s acknowledged prognostic value in mCRPC patients. Indeed, in this patient, the evidence of low FDG uptake was associated with NED, which is a known predictor of resistant and more aggressive disease [11]. On the other hand, it challenges the common assumption that mCRPC is systematically associated with increased glycolysis in the presence of NED [12,13,14,15]. This is relevant given the emerging use of 177Lu-PSMA therapy in mCRPC. Indeed, it has been shown that PC cells with NED do not express PSMA and are not affected by the 177Lu-PSMA therapeutic effect [16]. In order to lower the risk of costly and useless 177Lu-PSMA treatments, the use of 18F-FDG PET/CT imaging has been proposed to select patients for PSMA targeted therapy [17], even in the context of clinical trials [18]. However, the present observation suggests the eventual occurrence of low-FDG avid NED in mCRPC, in which this approach may be inadequate. This finding is coherent with the previous observations by Chen et al. [19] and Acar et al. [20]. Altogether, these data suggest that further studies are needed to explore the interplay between the 18F-FDG and somatostatin analogs signals in NED mCRPC patients. As a final consideration, functional imaging of somatostatin receptor expression may pave the way toward implementing novel targets for treating this aggressive subtype of PC through receptor-targeted chemo/irradiation interventions.

## Data Availability

Not applicable.

## References

[1] Jadvar H., Desai B., Ji L., Conti P.S., Dorff T.B., Groshen S.G., Pinski J.K., Quinn D.I. (2013). Baseline 18F-FDG PET/CT parameters as imaging biomarkers of overall survival in castrate-resistant metastatic prostate cancer. J. Nucl. Med..

[2] Bauckneht M., Capitanio S., Donegani M.I., Zanardi E., Miceli A., Murialdo R., Raffa S., Tomasello L., Vitti M., Cavo A. (2019). Role of Baseline and Post-Therapy 18F-FDG PET in the Prognostic Stratification of Metastatic Castration-Resistant Prostate Cancer (mCRPC) Patients Treated with Radium-223. Cancers.

[3] Bauckneht M., Rebuzzi S.E., Signori A., Donegani M.I., Murianni V., Miceli A., Borea R., Raffa S., Damassi A., Ponzano M. (2020). The Prognostic Role of Baseline Metabolic Tumor Burden and Systemic Inflammation Biomarkers in Metastatic Castration-Resistant Prostate Cancer Patients Treated with Radium-223: A Proof of Concept Study. Cancers.

[4] Bonkho H. (1998). Neuroendocrine cells in benign and malignant prostate tissue: Morphogenesis, proliferation, and androgen receptor status. Prostate Suppl..

[5] Palapattu G.S., Wu C., Silvers C.R., Martin H.B., Williams K., Salamone L., Bushnell T., Huang L.S., Yang Q., Huang J. (2009). Selective expression of CD44, a putative prostate cancer stem cell marker, in neuroendocrine tumor cells of human prostate cancer. Prostate.

[6] Zou M., Toivanen R., Mitrofanova A., Floch N., Hayati S., Sun Y., Le Magnen C., Chester D., Mostaghel E.A., Califano A. (2017). Transdierentiation as a Mechanism of Treatment Resistance in a Mouse Model of Castration-Resistant Prostate Cancer. Cancer Discov..

[7] Patel G.K., Chugh N., Tripathi M. (2019). Neuroendocrine Differentiation of Prostate Cancer-An Intriguing Example of Tumor Evolution at Play. Cancers.

[8] Hvamstad T., Jordal A., Hekmat N., Paus E., Fosså S.D. (2003). Neuroendocrine serum tumour markers in hormone-resistant prostate cancer. Eur. Urol..

[9] Shimomura T., Kurauchi T., Sakanaka K., Kimura T., Egawa S. (2020). Clinical investigation of neuroendocrine differentiation in prostate cancer. J. Clin. Oncol..

[10] Spetsieris N., Boukovala M., Patsakis G., Alafis I., Efstathiou E. (2020). Neuroendocrine and Aggressive-Variant Prostate Cancer. Cancers.

[11] Deorah S., Rao M.B., Raman R., Gaitonde K., Donovan J.F. (2012). Survival of patients with small cell carcinoma of the prostate during 1973-2003: A population-based study. BJU Int..

[12] Choi S., Ettinger S.L., Lin D., Xue H., Ci X., Nabavi N., Bell R.H., Mo F., Gout P.W., Fleshner N.E. (2018). Targeting MCT 4 to reduce lactic acid secretion and glycolysis for treatment of neuroendocrine prostate cancer. Cancer Med..

[13] Li W., Cohen A., Sun Y., Squires J., Braas D., Graeber T.G., Du L., Li G., Li Z., Xu X. (2016). The role of CD44 in glucose metabolism in prostatic small cell neuroendocrine carcinoma. Mol. Cancer Res..

[14] Bakht M.K., Lovnicki J.M., Tubman J., Stringer K.F., Chiaramonte J., Reynolds M.R., Derecichei I., Ferraiuolo R.M., Fifield B.A., Lubanska D. (2020). Differential Expression of Glucose Transporters and Hexokinases in Prostate Cancer with a Neuroendocrine Gene Signature: A Mechanistic Perspective for 18F-FDG Imaging of PSMA-Suppressed Tumors. J. Nucl. Med..

[15] Liu Y. (2008). FDG PET-CT demonstration of metastatic neuroendocrine tumor of prostate. World J. Surg. Oncol..

[16] McBean R., O’Kane B., Parsons R., Wong D. (2019). Lu177-PSMA therapy for men with advanced prostate cancer: Initial 18 months experience at a single Australian tertiary institution. J. Med Imaging Radiat. Oncol..

[17] Parida G.K., Tripathy S., Datta Gupta S., Singhal A., Kumar R., Bal C., Shamim S.A. (2018). Adenocarcinoma Prostate With Neuroendocrine Differentiation: Potential Utility of 18F-FDG PET/CT and 68Ga-DOTANOC PET/CT Over 68Ga-PSMA PET/CT. Clin. Nucl. Med..

[18] Hofman M.S., Emmett L., Sandhu S., Iravani A., Joshua A.M., Goh J.C., Pattison D.A., Tan T.H., Kirkwood I.D., Ng S. (2021). Trial Investigators and the Australian and New Zealand Urogenital and Prostate Cancer Trials Group. [177Lu]Lu-PSMA-617 versus cabazitaxel in patients with metastatic castration-resistant prostate cancer (TheraP): A randomised, open-label, phase 2 trial. Lancet.

[19] Chen S., Cheung S.K., Wong K.N., Wong K.K., Ho C.L. (2016). 68Ga-DOTATOC and 68Ga-PSMA PET/CT Unmasked a Case of Prostate Cancer with Neuroendocrine Differentiation. Clin. Nucl. Med..

[20] Acar E., Kaya G.Ç. (2019). 18F-FDG, 68Ga-DOTATATE and 68Ga-PSMA Positive Metastatic Large Cell Neuroendocrine Prostate Tumor. Clin. Nucl. Med..

